# Building capacity for HIV and implementation science among students in the United States: the stimulating training and access to HIV research experiences (STAR) program

**DOI:** 10.3389/fpubh.2025.1637752

**Published:** 2025-10-14

**Authors:** Ucheoma Nwaozuru, Joseph D. Tucker, Idia B. Thurston, Collins O. Airhihenbuwa, Rhonda BeLue, Weiming Tang, Chisom Obiezu-Umeh, Onyekachukwu Anikamadu, Khadijah Ameen, Christian Herrara, Alexis Engelhart, Tochukwu Patrick, Ujunwa Onyeama, David Oladele, Bryce Puesta Takenaka, Olufunto A. Olusanya, Temitope Ojo, Juliet Iwelunmor

**Affiliations:** ^1^Department of Implementation Science, Wake Forest University School of Medicine, Winston-Salem, NC, United States; ^2^Institute for Global Health and Infectious Diseases, University of North Carolina at Chapel Hill, Chapel Hill, NC, United States; ^3^Clinical Research Department, Faculty of Infectious and Tropical Diseases, London School of Hygiene and Tropical Medicine, London, United Kingdom; ^4^Bouvé College of Health Sciences, Institute for Health Equity and Social Justice Research, Northeastern University, Boston, MA, United States; ^5^School of Public Health, Georgia State University, Atlanta, GA, United States; ^6^College for Health Community and Policy, University of Texas at San Antonio, San Antonio, TX, United States; ^7^Department of Medical Social Sciences, Center for Dissemination and Implementation Science, Northwestern University Feinberg School of Medicine, Chicago, IL, United States; ^8^Brown School of Social Work, Washington University in St. Louis, St. Louis, MO, United States; ^9^Department of Psychological Sciences, University of Missouri, Columbia, MO, United States; ^10^Division of Infectious Diseases, Washington University School of Medicine in St. Louis, St. Louis, MO, United States; ^11^College for Public Health and Social Justice, Saint Louis University, St. Louis, MO, United States; ^12^Clinical Sciences Department, Nigerian Institute of Medical Research, Lagos, Nigeria; ^13^Department of Social and Behavioral Sciences, Yale School of Public Health, New Haven, CT, United States

**Keywords:** capacity-building, implementation science, HIV, participatory approaches, mentorship

## Abstract

**Background:**

Expanding HIV research capacity among the global majority (individuals identifying as Black/African American, American Indian and Alaska Native, Asian, Native Hawaiian and Other Pacific Islander, Multiracial, and Hispanic/Latino) is important. However, achieving national goals to increase the pool of implementation science and HIV early-stage investigators from underrepresented backgrounds remains elusive, largely due to limited investment in training and mentoring these individuals. To address this issue, we launched the Stimulating Training and Access to HIV Research Experiences (STAR) program, a partnership led by Saint Louis University and the University of North Carolina at Chapel Hill in collaboration with Georgia State University and Texas A&M University. The STAR program aims to establish a pathway for Underrepresented minority (UREM) students to engage in HIV and implementation science research.

**Methods:**

We launched a crowdsourcing open call from November 30, 2022, to January 22, 2023, to identify potential trainees at the four participating institutions (Prompt: “How might we promote HIV prevention among youth aged 13–24 years in your community?”). The finalists from the crowdsourcing call participated in a 2-day designathon, which included didactic introductory lectures on HIV, dissemination and implementation science. The finalists participated in a 6-week innovation bootcamp, including modules on HIV research, implementation science, research ethics, and fieldwork experience with community partners. We assessed the acceptability of the STAR program through participant self-reported surveys on their experience and evaluation of the lectures.

**Findings:**

Twenty-four individuals applied to the STAR program by completing the crowdsourcing open call, 12 were selected for the designathon, and 10 completed the fellowship. The first cohort of STAR trainees (10 students—6 undergraduate and 4 graduate students) successfully completed the STAR innovation bootcamp. The innovation bootcamp culminated in seven proposals that the trainees implemented and evaluated over 12 months, with support from the research team, mentors, and participatory learning community. The implementation strategies proposed by the trainees include the use of peer engagement, storytelling, digital engagement tools, and artificial intelligence to promote awareness of HIV and increase the uptake of HIV testing. All the participants were satisfied with the STAR program (90% very satisfied and 10% satisfied) and indicated enthusiasm for pursuing academic and research careers in HIV and/or implementation science.

**Conclusion:**

Building a pathway for UREM investigators is crucial to ending the HIV epidemic. The STAR program may enhance interest, build research capacity, and increase the UREM talent pool retained in this field.

## Background

People of the global majority (individuals identifying as Black/African American, American Indian and Alaska Native, Asian, Native Hawaiian and Other Pacific Islander, Multiracial, and Hispanic/Latino) in the United States make up a significant proportion of newly diagnosed individuals with HIV (1, 2). The proportion of the global majority newly diagnosed with HIV is inversely correlated to the global majority workforce or researchers in the HIV field (1, 2). Racial and ethnic inequities persist along the HIV prevention and care continuum - poor indicators for HIV testing, HIV prevention, linkage to and retention in care, antiretroviral therapy (ART) uptake and adherence, and viral suppression (3). While evidence-based interventions (EBIs) and strategies exist to address these issues, the problem lies in individual, structural, and social barriers that impede their uptake and reach (4).

The field of dissemination and implementation (D&I) science can potentially reduce these translational gaps, but it requires transformative approaches that are community-engaged, equitable, and reflective of the needs of individuals most affected by the problem—i.e., people from the global majority (5). Therefore, it is especially important to have a workforce and researchers who are representative of the communities most affected by HIV. The current workforce does not mirror that. The number of investigators who identify as the global majority in biomedical research, including HIV, is suboptimal (1, 6–8). A well-trained and diverse pool of researchers represents a crucial component towards equitable implementation and addressing the persistent disparities along the HIV prevention and care continuum (7, 9).

The global majority, comprising over 30% of the US population, accounts for less than 9% of individuals in health and biomedical professions (10). Underrepresented groups are a critical resource of talent that could be nurtured to expand the HIV research workforce and elucidate cultural assets and resources for HIV prevention/care that may not be accessible to individuals outside the community (7, 11). HIV research led by investigators who are part of the affected community offers an opportunity to understand cultural and contextual factors that can enhance the utility of the research work (7). Therefore, it is important that the research space is diverse and includes individuals of various backgrounds. However, existing training programs are limited, and few academic and research institutions have innovative skills development, research experiences, and mentoring activities to support high-quality HIV training for racial and ethnic minorities (9).

To address this issue, we launched the Stimulating Training and Access to HIV Research Experiences (STAR) program, funded by the National Institute of Allergy and Infectious Diseases (NIAID). The program is a partnership led by Saint Louis University and the University of North Carolina at Chapel Hill, in collaboration with Georgia State University and Texas A&M University. The protocol manuscript is currently under journal review. The STAR program aims to establish a pathway to increase entry into and retention of trainees in HIV and D&I research, particularly those from backgrounds underrepresented in biomedical research. STAR incorporates elements of participatory action research to provide hands-on HIV research experience, skills development, and mentoring opportunities to undergraduate and graduate students. This manuscript describes the STAR program’s structure and core components, as well as its impact on trainees’ self-reported scientific proficiency and project outcomes.

## Methods

The STAR program used a multi-phase approach to recruit and train scholars. This included a crowdsourcing open call, a designathon, and an innovation bootcamp.

### Eligibility criteria

Students who identified as underrepresented minorities from the four participating institutions (Saint Louis University, University of North Carolina at Chapel-Hill, Georgia State University, and Texas A & M University) were eligible to participate in the STAR program. Undergraduate and graduate students were eligible to participate in the program. Participants had the option to apply as individuals or teams. The application to the STAR program included completion of a crowdsourcing open call application packet, which included a response to the crowdsourcing open call prompt, demographics information, resume/CV, and transcripts.

### The crowdsourcing open call

Crowdsourcing is a process whereby a group of people attempt to solve all or part of a problem and then shares their solutions with the communities of interest (12, 13). Crowdsourcing takes a bottom-up approach for problem-solving, and has been successfully used to solicit innovative ideas in several areas, including developing strategies to promote the uptake of HIV testing (14–16), antimicrobial drug discovery (17), and STI testing (18). The STAR crowdsourcing open call aimed to identify STAR participants and provide an opportunity for them to develop HIV prevention research ideas. The crowdsourcing open call was launched on November 30th, 2022, through January 22nd, 2023. We disseminated the open call via flyers on social media, direct emails to professors in relevant programs at the participating institutions, webinar events, and announcements by campus liaisons. The campus liaisons were student representatives from participating institutions who acted as intermediaries between the program leaders and the STAR participants. They assisted with participant recruitment, coordinated program activities such as designathons and bootcamps, scheduled meetings, and supported the development and implementation of participants’ ideas. The open call entry required: (a) response to the prompt—*“How might we promote HIV prevention services among youth aged 14–24 years old in your community? We are particularly interested in communities with populations who are Black, Latine, Asian, Brown, Indigenous, and/or dual- or multi-heritage, also known as the global majority (Response must not exceed 500 words)”*; (b) personal statement; (c) CV or Resume; and (d) faculty letter of recommendation (at least one and at most two). Each category was scored on a scale from 0 to 5 points. The open call question was scored using the following criteria: (1) clear and concise description; (2) relevance; (3) novelty; (4) feasibility, scalability/ replicability, and sustainability; and (5) promotion of equity and fairness, with 5 being the highest possible score. The scores were also weighed, with the Open Call Response worth 50%, the Personal Statement 20%, the CV/Resume 15%, and the Letter of Recommendation 15%.

### The virtual designathon

A designathon is a three-step process informed by design thinking that includes *preparation with end-users and others* (open call for ideas to engage end-users and other key individuals to identify ideas to prepare for collaboration), *intensive collaboration* (interaction between participants and mentors to foster cross-disciplinary problem-solving and refinement of team ideas), and *follow-up activities for implementation and research* (plans for implementation of solutions beyond designathons, mentorship for participants to support implementation, and plans for monitoring and evaluation) (19). A systematic review of designathons provides evidence for the effectiveness of this approach (20). The STAR designathon was hosted virtually from February 17 to 19, 2023. Top entries (*N* = 12) from the crowdsourcing open call were invited to participate in the designathon to further develop their ideas. The designathon used a workshop-style format where participants learned about design-thinking concepts, such as rapid prototyping and co-creation, to strengthen their ideas. At the end of the designathon, participants pitched their solutions to an expert panel of 5 judges. The panel of judges consisted of public health and implementation science researchers, practitioners, and representatives from community organizations. The contest question for the designathon was similar to the open call: “How *might we promote HIV prevention services among youth aged 14–24 in your community?”* At the end of the designathon, the participants presented three key deliverables: (1) a PLAN (People, Learning, Adapting, Nurturing) (21) on how to engage and sustain engagement with their community partners; (2) A specific aims page introducing their solution, main objective, and potential impact, and (3) a 5- min pitch, which were evaluated by the judges based on these five criteria: (a) clear and concise description; (b) relevance; (c) novelty; (d) feasibility, scalability/replicability, and sustainability; and (e) promotion of equity and fairness.

### The hybrid innovation bootcamp

Following the designathon, the STAR cohort participated in a 6-week hybrid summer innovation bootcamp. An innovation boot camp is an accelerated training program designed to build capacity for implementing solutions and typically follows a designathon to provide participants with research and project strategy skills (22). The bootcamp comprised of 3-weeks synchronous and asynchronous sessions, 2 weeks of fieldwork at a collaborating community partner organization (during this phase, the scholars gained feedback on their ideas from their community partners. It was also an opportunity to understand the feasibility of implementing their proposed solutions in collaboration with the community partners), and 1 week of in-person activities.

The program was framed to build general knowledge of HIV/AIDS and D&I and cross-cutting topics such as practices in grant preparation, community participatory research, JEDI (justice, equity, diversity, and inclusion), and leadership principles among the STAR fellows. A compressed curriculum for the innovation bootcamp is shown in Table 1. At the end of the innovation bootcamp, the participants presented three key deliverables: a project protocol that included a description of the solution, significance, innovation, and proposed implementation approach, a project PLAN (21) on how to engage and sustain engagement with their community partner for the proposed solution, and a 10-min pitch presentation. The bootcamp culminated with participants pitching their final ideas to a panel of six expert judges during the final week of in-person activities. The judges comprised public health and implementation science researchers, practitioners, and representatives from community organizations. The ideas were judged based on the 5 criteria that were used in the designathon phase: (a) clear and concise description; (b) relevance; (c) novelty; (d) feasibility, scalability/replicability, and sustainability; and (e) promotion of equity and fairness.

**Table 1:** Abridged version of the STAR innovation bootcamp curriculum.

Weeks	Activities
Week 1 [Monday–Friday]	Overview of implementation science Introduction to dissemination and implementation science theories, models, and frameworks Landscape of HIV/AIDS research among minority youth population and social determinants of health Participatory approaches to research I Panel discussion [Community-based HIV research, professional development series] Discourse reflections [Application of the PEN-3 cultural model] Conversation café [Introduction to professional development, Team ideation]
Week 2 [Tuesday–Friday]	Introduction to qualitative research Introduction to grant writing [Part 1 & 2] MHealth in HIV research Health equity in HIV research Participatory approaches to research II Conversation café [Decision-making, community organizational mapping] Discourse reflections [Application of the PEN-3 cultural model on creative decision-making for HIV prevention programs, Mapping community organizations’ priorities, needs, assets, values & hurdles] Mixed-methods approach and evaluation Panel discussion [Youth panel]
Week 3 [Monday–Friday]	Skills-building on leadership [Part 1 & 2] Skills building Justice, Equity, Diversity, and Inclusion [Part 1 & 2] Ethics of youth engagement in HIV/AIDS research Basic research on the cost and economic evaluation of research Conversation café [Identifying leverage and constraints with HIV/AIDS research and young people of color at the state and community level, public speaking for change]
Weeks 4 & 5	Fieldwork and community engagement activities (locally)
Week 6	In-person STAR program

The weekly activities included daily reflections on the prior day’s activities and dedicated time for team activities related to their STAR deliverables.

### Follow-up activities

Beyond the bootcamp, the STAR scholars had access to their faculty mentors at their respective institutions and the participatory learning community. The participatory learning community was designed to be a collaborative space for STAR scholars to share progress on their pilot work, and brainstorm challenges with implementing their solutions. In addition, we held quarterly virtual meetings with the STAR scholars; this was an opportunity to share updates on the work and get feedback from their peers and program faculty. Outside the STAR-wide meetings, the campus liaisons and institution directors held periodic meetings with the scholars.

### Data collection

Feedback on the STAR bootcamp was obtained through surveys designed to collect quantitative and qualitative responses before and after the innovation bootcamp related to the fellow’s overall experience. The questions were related to their experience with the program logistics, program faculty, presentations, didactic sessions, and recommendations for improvement. Closed-ended questions consisted of five-point Likert-type scales from “very dissatisfied” to “strongly satisfied.” At the beginning of the bootcamp, participants were provided with a survey to rate their knowledge of the core competencies of the program: (a) dissemination and implementation science, (b) clinical sciences, (c) leadership, and (d) Justice, Equity, Diversity, and Inclusion (JEDI), on a 5-point Likert scale (1 = strongly disagree, 5 = strongly agree). The implementation science competencies were informed by the work of Padek and colleagues (23). The survey included questions related to participants’ skills and competencies in the topic areas, including definitions of key terminologies in the field, guiding theories and approaches, methods, designs, and analysis, and practice-based considerations (23). The participants completed the same questions at the end of the innovation bootcamp. Open-ended questions gathered feedback on participants’ satisfaction with the program, perceptions of its components and logistics, mentorship opportunities, networking experiences, learning outcomes, and program delivery and organization.

### Analysis

#### Quantitative data

Demographic data collected from participants during the crowdsourcing open call, designathon, and bootcamp phases of the program, including their age, sex, race, ethnicity, level in school, and institution affiliation, were compiled and analyzed using descriptive statistics (frequencies and proportions). For Likert scale questions, the frequencies of the responses were calculated. To compare changes from the pretest to the posttest at the bootcamp, paired sample *t-tests* with Cohen’s *d* effect sizes were used. A *p*-value <0.05 was considered statistically significant. All analyses were performed using SPSS (version 22; IBM Corp).

#### Qualitative data

##### Data from the crowdsourcing open call and designathon

The qualitative data from the crowdsourcing open call and designathon were deidentified for analysis. A thematic analysis was conducted using open coding, which assigns themes to capture specific ideas, and axial coding, which explores linkages between concepts and categories and determines common themes (24). The thematic analysis involved two members of the team (COU and UN) initially reading through the data to familiarize themselves with the responses, after which they extracted texts to generate a codebook that identified recurring categories and themes across the data set independently. The two coders (COU and UN) then compared, discussed, and synthesized their coding process, which was merged into the final codebook. The two coders then tested the codebook against three submissions, made revisions, and resolved discrepancies before moving to the stage of complete coding. All submissions were then characterized using the codebook, and overarching categories were closely examined to identify analytic themes. *Qualitative Survey data*: Due to the exploratory nature of the open-ended questions included in the survey, we analyzed the text responses using an inductive thematic approach (25, 26). One member of the team (COU) collated the responses to the open-ended questions for data cleaning and quality checks. Following this, two members of the research team (UN and COU) independently read the texts to become familiar with the data before developing codes. Then the open-ended questions were manually coded independently by two members of the team (UN and COU) to determine emerging themes. The two reviewers compared their themes for consistency, and differences were resolved by consensus. The findings are organized based on emerging themes and corresponding quotations from written open-ended responses.

### Ethics

This study was determined to be non-human subjects research by the Saint Louis University Review Board.

## Results

### Crowdsourcing open-call

We received 24 fully completed submissions (SLU = 9; TAMU = 6; GSU = 5; and UNC = 4). The majority of the entries were from individuals who identified as women (56.5%) and Black or African-American (50.0%). The mean age of applicants was 24.5 years. Table 2 provides the demographics of eligible submissions to the crowdsourcing open call and details of who progressed on to the designathon and bootcamp phases.

**Table 2:** Demographics of STAR participants.

Measures	Crowdsourcing open call (*N* = 24)	Designathon (*N* = 12)	Innovation bootcamp (*N* = 10)
	Overall *n* (%)	Overall *n* (%)	Overall *n* (%)
Sex			
Female	15 (56.5)	8 (66.7)	8 (80.0)
Male	9 (37.5)	4 (33.3)	2 (20.0)
Age			
18–24	12 (50.0)	7 (63.6)	6 (60.0)
25–31	9 (37.5)	3 (27.3)	3 (30.0)
32–38	3 (12.5)	1 (9.10)	1 (10.0)
Race			
Black or African-American	12 (50.0)	5 (45.5)	6 (60.0)
Asian	9 (37.5)	4 (36.4)	4 (40.0)
White/Caucasian	3 (12.5)	1 (9.1)	
Ethnicity			
Hispanic/Latino	1 (4.2)	1 (9.1)	_
School level			
Undergraduate	12 (50.0)	7 (63.6)	6 (60.0)
Graduate	12 (50.0)	4 (36.4)	4 (40.0)
University			
SLU	9 (37.5)	4 (36.4)	3 (30.0)
TAMU	6 (25.0)	2 (18.2)	2 (20.0)
GSU	5 (20.8)	3 (27.3)	3 (30.0)
UNC	4 (16.7)	2 (18.2)	2 (20.0)

SLU, Saint Louis University; TAMU, Texas A&M University; GSU, Georgia State University; UNC, University of North Carolina at Chapel Hill; STAR Stimulating Training and Access to HIV Research Experiences. Age (*N* = 11), School level (*N* = 11), University (*N* = 11).

In addition to providing strategies for promoting HIV prevention services among youth in their communities, 54% (*n* = 13) of the entries outlined barriers to HIV prevention. The key barriers to the uptake of HIV preventive services include (a) Limited access to comprehensive HIV education and materials, (b) Stigma and misconceptions that prevent open conversations about HIV or accessing necessary preventive services, (c) Structural barriers such as poverty, discrimination, and violence that may impede access to healthcare services, (d) Political determinants that influence the availability of sexual and reproductive knowledge and services, and (e) Limited youth-friendly strategies. Conventional methods of programming and promotion of HIV knowledge and information may not be engaging and appealing to youth. See Appendix 1 for barriers to the uptake of HIV prevention services emerging from the crowdsourcing open call.

Themes from the crowdsourcing open call entry on strategies to promote HIV prevention services among youth aged 14–24 years old in the respondents’ communities included: (a) Use of storytelling to make the information relatable to youth, (b) Use of social media and digital technologies for campaigns and dissemination of accurate and reliable HIV information, (c) Use of competitions and incentive-driven programs, (d) Youth engagement in program delivery-engaging youth as peer navigators, champions, or implementors of HIV prevention programs for youth, and (e) Partnering with existing organizations that serve youth, such as youth community-based organizations, student clubs, and after-school programs to deliver HIV prevention programs for youth. See Appendix 2 for the emerging themes on strategies to promote HIV prevention among youth aged 14–24 years from the crowdsourcing open call.

### Designathon

We selected 12 participants from the open call to move on to the designathon. After 2 days of strengthening their solutions and a pitch presentation to the judging panel, all participants were selected to join the STAR innovation boot camp (see Table 2).

Themes from the solutions on strategies to promote HIV prevention services among youth aged 14–24 years in their respondents’ communities included: (a) promoting awareness and education on HIV prevention through youth engagement. This included utilizing sex-positive approaches, utilizing art to foster engagement and appeal among youth, and using interactive and informational videos for education. (b) Partnership with youth-serving organizations and youth in implementing HIV services. This included collaborations with schools, after-school programs, and community-based organizations to deliver HIV programs. In addition, utilizing peer-to-peer delivery of HIV services or for health promotion, (c) building trust among youth and addressing stigma. This included fostering conversations in safe spaces and (d) using social media and digital technology. This included leveraging social media and digital technologies such as websites and software applications to deliver HIV prevention information and for demand creation. Additional information about the solutions at the designathon is provided in Appendix 3. The teams from the designathon were then invited to join the innovation bootcamp to build on their implementation pilot solutions.

### STAR innovation bootcamp

Ten out of the twelve participants from the designathon participated in the STAR bootcamp as STAR scholars (Table 2 provides demographics). The other two participants from the designathon could not proceed to the bootcamp due to scheduling conflicts. Most of the STAR first cohort were undergraduate students (60%).

### Emerging pilot projects

The STAR bootcamp culminated in seven proposals led by the STAR scholars, with support from the research team, mentors, and participatory learning community engagements. The various teams proposed diverse strategies to promote HIV prevention and awareness and address stigma among youth and minoritized populations. Most of the solutions involved some community-engagement components through activities such as listening sessions with a community interested in learning about their needs, resources, and assets; peer engagement through youth advisory boards; crowdsourcing open calls to generate innovative and creative solutions from young people for HIV prevention; art exhibits; and storytelling. In addition, technology-driven solutions were also suggested, including leveraging machine learning for risk assessment and tailoring of health information, using social media campaigns, gamified platforms for HIV education, and an interactive website for the geolocation of youth-friendly services for HIV. Overall, the solutions focused on some HIV prevention objectives, including reducing HIV stigma, promoting HIV knowledge, or the uptake of preventive services such as HIV testing and PrEP. The proposed solutions by the STAR scholars are provided in Table 3. Judges scores of the teams’ solutions are provided in Appendix 4.

**Table 3:** The description of the proposed team solutions.

Solution name	Team composition	Description	Audience of interest [age, location]	Proposed project objectives
ATL in ATL: Advocating, Teaching, and Leading HIV & AIDS awareness for Black women in Atlanta	3 Team Members (3 Females)	A multi-component solution comprising an in-person listening session to understand the needs and assets among the intended audience, an Instagram campaign for HIV awareness, and an in-person launch event on Georgia State University’s campus in partnership with student health and local collaboration resources (SisterLove & BLKHLTH).	Black women at Georgia State University, Atlanta, Georgia, aged 18–24 years	Promote open and safe conversation among young Black women on HIV Promote uptake of HIV prevention services among young Black women
HIVE: The ART of Coming Together	1 Team Member (1 Female)	A community-engaged approach comprising: (a) Focus group discussions with St. Louis Agency on Training and Employment (SLATE) and creating a youth advisory board, (b) A HIV informational website, and (c) community partnership and impact assessment.	Adolescents and young adults in the City of St. Louis, Missouri, aged 13–24 years	To promote the uptake of evidence-based HIV services To provide HIV prevention and care knowledge
Living Reality	1 Team Member (1 Female)	A peer-to-peer storytelling and mentoring approach to promote HIV knowledge, preventive services, and address stigma. Mentors (young people) will share their journeys and experiences living with HIV through videos, narratives, poetry, visual arts, or any form the mentor feels comfortable with.	University Students at Saint Louis University, Saint Louis, Missouri, aged 18–24 years	To increase HIV testing To increase condom use To increase knowledge about HIV/AIDS and safe sex practices To address stigma and create a safe environment
NULAGE: New Understanding & Learning in AIDS and Gender Education	1 Team Member (1 Female)	An interactive website platform that provides information focused on addressing HIV misconceptions and myths with gamification capabilities, a location for youth-friendly health services for HIV and other preventive services, and Google Maps integrations to allow the identification of services.	Young people in Bryan/College Station, Texas, aged 18–19 years	To address HIV misconceptions and stigma
Project angels	1 Team Member (1 Female)	Develop and implement a crowdsourcing open call focused on HIV prevention to foster creativity and self-expression among youth by allowing them to create artwork individually or as a team through dance, painting, video creation, drama, and other art forms. In addition, the piece of art will be displayed at the Williams and Associates open-house art exhibit to promote community engagement and conversations related to HIV prevention.	African American youth in Saint Louis City, aged 13–24 years	Raise awareness of HIV
Project SPARK: Strengthening Peer-led Advocacy for Resilience Knowledge in HIV Prevention	1 Team Member (1 Male)	The solution leverages an evidence-based intervention, *“Prime Time,”* which was effective in promoting positive sexual health behaviors among sexually active adolescent females. The proposed Project Spark would include educational sessions delivered in a workshop format focused on promoting HIV self-testing and advocacy for PrEP uptake. The solution would be to delivered over five sessions, weekly for 45 min, in collaboration with the Boys and Girls Club at Bryan, Texas.	Racial/ethnic minority Bryan/College Station, Texas, aged 13–18 years	To increase HIV self-testing To promote awareness of Pre-exposure prophylaxis (PrEP)
Tech and Media Leverage for PrEP uptake among young men who have sex with men (MSM) of color	2 Team Members (1 Female & 1 Male)	An integrated risk assessment and decision-making tool created by the community for the community. The tool will leverage machine learning, a gamified digital platform with interactive elements, co-creation with the community, and a human-centered design approach to promote information about HIV prevention.	MSM of Color, Los Angeles, California, aged 18–24 years	To increase the number of young MSM of color who initiate PrEP and improve retention

ATL, Atlanta; PrEP, Pre-exposure prophylaxis; MSM, men who have sex with men.

### STAR bootcamp evaluation

#### Quantitative evaluation

A pre-post assessment of the core competencies of the STAR program showed an overall improvement in the four areas: (a) dissemination and implementation science (D &I), (b) clinical sciences, (c) leadership, and (d) JEDI. However, only the increase in D&I and clinical sciences knowledge was statistically significant. The difference in D & I knowledge at baseline and the completion of the boot camp had a large effect size (Cohen’s *d* = 0.99). The difference in Clinical sciences knowledge at baseline and the completion of the boot camp had a moderate effect size (Cohen’s *d* = 0.74). Table 4 provides a summary of this assessment.

**Table 4:** Pre-post assessment of STAR core competencies.

Competencies	Mean and standard deviation		Mean difference and Cohen’s D		
	Pre (Baseline)	Post (6-week)	Mean difference	*p-*value	Cohen’s D
D&I	2.56 ± 1.00	4.13 ± 0.63	+1.57	<0.001*	0.998
Clinical Sciences	3.64 ± 0.83	4.51 ± 0.53	+ 0.87	0.005*	0.737
Leadership	4.20 ± 0.92	4.65 ± 0.474	+ 0.45	0.215	1.066
JEDI	4.86 ± 0.32	4.85 ± 0.21	+ 0.01	0.764	0.136

D&I, dissemination and implementation science; JEDI, justice, equity, diversity, and inclusion.

In general, 90% of the participants were “very satisfied” with the STAR program, and 10% were “satisfied.” Further, 90% of the participants indicated they would recommend the STAR program to their peers. Regarding the mentorship experience, most of the STAR scholars found the support from their peers helpful (80%), and 90% indicated that the feedback from their faculty on their ideas and the final project was helpful, respectively. Additional reports are provided in Appendix 5.

#### Qualitative evaluation

A qualitative evaluation of the innovation bootcamp among the STAR scholars shows an overall positive experience with the STAR program and its contents. In addition, the scholars highlighted some areas for improvement in the boot camp experience. Three main qualitative themes emerged from our analysis of the scholars’ responses to open-ended questions on pre- and post-program evaluation surveys: (a) STAR boot camp feedback. This included feedback on overall satisfaction with the program, program components and logistics, networking, and mentoring components, (b) feedback on course components, and (c) some challenges experienced and areas of improvement. This included some challenges experienced in program delivery and organization, teamwork, dynamics, and course durations. The emerging themes and corresponding sub-themes, where applicable, and quotes from the written feedback on the survey are provided in Table 5.

**Table 5:** Qualitative feedback on the STAR program.

Themes	Sub-themes (where applicable)	Exemplary quotes from written feedback
STAR Program Feedback	Overall Satisfaction	*“The challenge of it! The novelty of creating and self-presentation is incredible. I also loved the flexibility in our own creativity.”* *“I had an overall very positive experience this past weekend! Thank you to the whole team and everyone who was a part of STAR!”*
	Program components and logistics	*“The STAR provides a variety of resources to complete successful deliverables.”* *“I did wish everyone had a chance to share what they discussed in the breakout rooms. I understand that it was a factor of time, but I felt like I and my breakout partner shared a lot of great experiences, but also felt differently about some of them.”* *“The food from local restaurants, the welcoming atmosphere, and constant encouragement from all faculty made this an unforgettable experience not only for my career but likely for life.”*
	Networking	*“The positive aspects were meeting new people, being inspired, learning more about research, and exploring a new city.”* *“Getting to meet everyone in person. The great speakers we had. Receiving feedback on our project.”*
	*Mentorship*	*“Being able to talk through my idea with the mentors. They all had great advice, and it really helped shape my idea and presentation.”* *“I believe the most helpful component is having our mentors guide us throughout the program.”*
Feedback on course components	Relevance of the lectures	*“I really liked the guest lectures. We would never have access to such people otherwise.”* *“The lectures from the guest speakers and their openness into discussing their obstacles and how they overcame those was very helpful.”* *“Learning from various speakers and being able to connect with them, also being able to shine light on our aspirations outside of academia.”* *“The class was probably the most interactive, and it is probably one of the best sessions in the ongoing bootcamp”*
Challenges and areas of improvement	Program delivery and organization	*“An earlier start to the community engagement fieldwork; Thank you”!* *“It would be helpful to begin activities or be aware of potential activities soon after the designathon.”* *I feel that more individual hand holding and one on one engagement would have helped.”*
	Teamwork and dynamics	*“One thing I struggled with during the in-person portion of the bootcamp was collaborating effectively with my partner on the project. I felt like my ideas and contributions were subsumed in my partner’s. I understand that this is a potential hazard of group work. For future STAR scholars, I would suggest integrating some strategies to navigate group dynamics, specifically for the project, perhaps even for future careers as another aspect of professional development.”*
	Duration	*“I think some of the lectures went on a little too long.”* *“It was difficult to do other things because I was constantly on zoom.”* *“Some of the discourse assignments felt overwhelming in addition to our working on our proposals.”* *“I also did not like the time that was spent navigating the NIH website. Screenshots could have been taken in advance to avoid all the clunkiness.”*

## Discussion

We report on the first year of the implementation of the STAR program. The program seeks to support the development of HIV and D&I research skills among students to increase the pipeline of underrepresented researchers focused on HIV and D&I research. The STAR program utilized an innovative multi-phase, participatory approach to recruiting and training scholars to develop solutions for promoting HIV prevention services among youth aged 14–24 years old. The success of the STAR program was evident from feedback from the STARs. The program evaluation suggests overall satisfaction and acceptability among the STAR scholars. The participants valued peer interactions and the support from the STAR faculty and mentors. The use of participatory approaches, such as crowdsourcing and designathons, also expands the literature by providing innovative strategies for recruiting and engaging individuals in training.

The evaluation of the core competencies of the STAR program indicated gains by the end of the bootcamp. However, only the gains in D&I (e.g., lectures on D&I theories, methods, and frameworks) and clinical sciences (e.g., lectures on research study design and methodologies, identifying and measuring clinically relevant outcomes, and community engagement in research) components were statistically significant. These findings are congruent with other training programs for students who reported gains in research skills and academic knowledge (27). This highlights the value of the STAR program in enriching student content knowledge and research skills. Notably, leadership and JEDI areas were the highest rated at pretest; it is possible that a ceiling effect impacted the lack of change in those areas. Future iterations of STAR may want to improve the pretest measure or deepen skills in these areas since participants joined with such high knowledge at the outset.

Beyond the research and course materials, the program provided scholars with experiential research opportunities with the community partners they intended to work with. The scholars received robust research experience in idea/solution conceptualization and community engagement through implementation. The scholars learned the importance of community engagement and a strength-based approach to intervention, development, and implementation through this process. By centering community engagement and strength-based inquiry through the lecture format and the assignments, the STAR program cultivated and reinforced the capabilities and strengths of young people and communities toward leading an HIV-free generation (28). This builds on the consideration for inclusive co-creation of knowledge and strategies in D &I to improve health and create transformational change in systems that influence health (29).

At the end of the bootcamp, the scholars developed seven proposals to be implemented within their respective communities. The solutions generated by the scholars included community-engagement components to create demand for and promote the uptake of HIV prevention services among young people. Engaging communities to support HIV prevention research has been highlighted as critical to developing robust and locally relevant strategies (30). The strategies developed by the participants may have significant implications for designing HIV prevention interventions focused on elevating youth assets, leveraging digital technologies, building trust, and collaborating with existing organizations to optimize the delivery of youth-centered interventions.

Findings suggest that students and trainees can be involved in the co-production of knowledge and activities for HIV prevention in the early stage of their training. This can inform future training programs, fostering participatory strategies for engaging trainees and steering trainees towards developing competencies on centering people and communities in their research and projects.

Notably, mentorship was highlighted as an important aspect of the STAR program. This aligns with the WHO HEalth Research MEntorship in Low and Middle-Income CountrieS (HERMES) guide on institutionalizing research mentorship (31). The guide highlights the importance of supportive mentoring practices that elevate the strengths and capabilities of mentees. In addition, the high satisfaction with the multi-mentorship opportunity through peers and faculty members has been reported as an important attribute of training programs to enhance diversity and inclusion in research (32). Mentorship has been shown to be a very influential component of successful training experiences, career pathway development, and workforce development for trainees (32, 33).

Constructive feedback from the scholars revealed concerns about the lengthy nature of the online engagement component of the training during the 3 weeks of online lectures. While the online modality for lecture delivery was effective in engaging students in different locations simultaneously, there were some challenges with continually engaging the trainees for an extended period. This challenge is similar to other training programs that have utilized virtual platforms to deliver the training, which have shared concerns with continued engagement and retaining participants’ attention over an extended period of time (34). Future STAR trainings could reduce online time and increase active engagement with trainees through interactive activities. In addition, some of the participants indicated the need for an earlier engagement with their community partners to plan and implement their ideas. Looking forward to maximizing the impact of the STAR program, we will partner with community organizations from the onset to co-develop the open call prompt. This would ensure that solutions generated through the open call would be responsive to the immediate needs of community partners.

### Limitations

There are some limitations worth noting. One notable limitation is that there is a potential for selection bias. The participants were likely individuals already exposed to or interested in HIV or implementation science research. To minimize selection bias, we utilized several promotion strategies to enhance a wider reach, such as social media promotion, classroom announcements, and webinars. Nonetheless, involvement in training requires a level of interest in expanding their knowledge in the proposed area. This evaluation is based on an immediate assessment of the program. This is critical information to assess the program’s success in meeting the short-term goals. Future studies should follow students over time to assess the impact of the STAR program on their career and research trajectories. Lastly, reliance on self-reported measures may have introduced social desirability bias. Despite these limitations, the evaluation of the STAR program is highly acceptable among STAR scholars. The format of the STAR program can be replicated to improve core competencies on HIV and implementation science among undergraduate and graduate students, and develop a pathway for diverse researchers and professionals in the field.

Future directions for the STAR program include building community engagement and expanding the curriculum. Potential future curriculum topics include how to develop and implement demonstration projects within community organizations. We would incorporate novel strategies to keep participants engaged during the 3 weeks of lectures and participatory learning communities, such as including book clubs and journal clubs to introduce scholars to emerging research and topics in the field. In addition, given the challenges some participants faced with teamwork, the STAR curriculum and activities would include strategies to foster team cohesion and collaboration.

## Conclusion

In summary, findings from this work highlight the success of the STAR program in recruiting and training students in HIV and D&I research while centering the roles of community engagement, diversity, equity, and inclusion. This first iteration of the STAR program holds promise in fostering HIV research with an equity and implementation science lens among global majority scholars, which could help narrow gaps in health disparities in their respective communities. By training scholars who are underrepresented in the HIV field, we are nurturing the next generation of researchers and professionals who will contribute to innovation and excellence in HIV and D&I research. Partnering with community-based organizations and including fieldwork experiences elevated scholars’ experiences by allowing them to experience the real-world implementation of their solutions. The STAR participatory recruitment and training process could serve as an innovative model to foster interest and build research capacity, educational training, and mentorship for the next generation of HIV and D&I scientists.

## Data Availability

The raw data supporting the conclusions of this article will be made available by the authors, without undue reservation.
