# Supplementary material for: Building capacity for HIV and implementation science among students in the United States: the stimulating training and access to HIV research experiences (STAR) program
Source: Front Public Health. 2025 Oct 14;13:1637752. doi: 10.3389/fpubh.2025.1637752 (PMC12558973; doi:10.3389/fpubh.2025.1637752)
Supplement: Supplementary file 1 [file Data_Sheet_1.docx]

**Supplementary File**


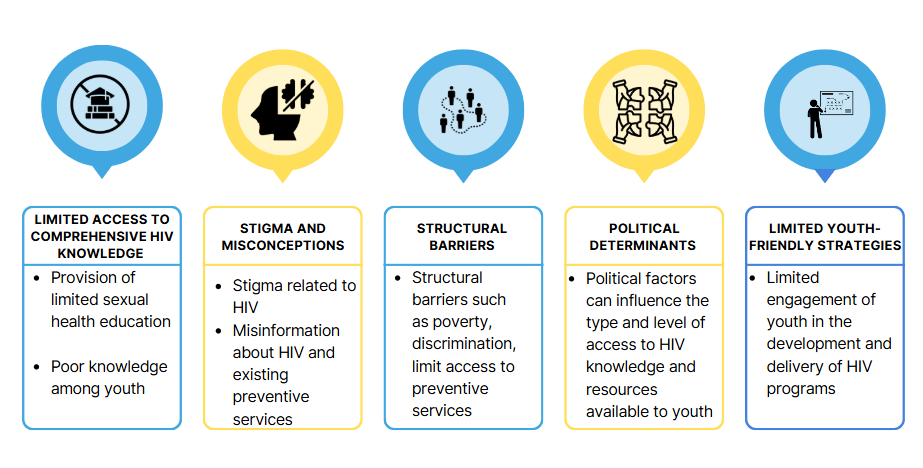
**Appendix 1.** Barriers to the uptake of HIV prevention services emerged from the crowdsourcing open call. [Image created on canva.com]


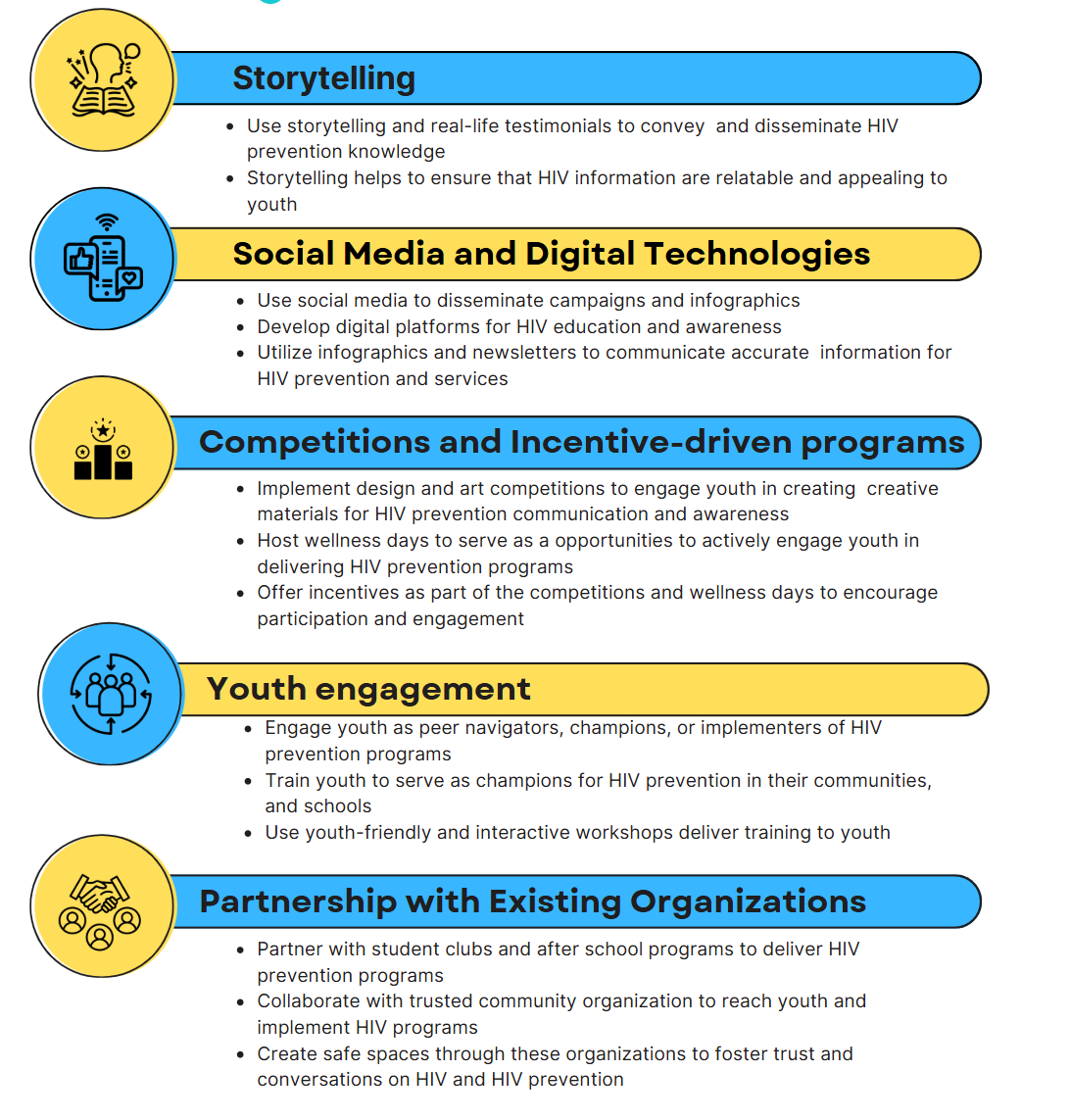
**Appendix 2.** Emerging themes on strategies to promote HIV prevention among youth aged 14-24 years from the crowdsourcing open call. [Figure created on canva.com

**Appendix 3. Description of the Team Solutions from the Designathon**

| **Solution Name** | **Focus Population** | **Solution Description and Goals** |
| --- | --- | --- |
| No Name at the Designathon | Young MSM of Color (18-24 years old) | Integrated risk-assessment shared decision-making tool, increasing uptake of PrEP in UREM MSM, and adapting evidence-based interventions. Bringing in human-centered design, deploying where the community is, Adapting to different communities and languages |
| The H-App | Youth in St. Louis | The H-APP, Promoting Privacy, Protecting users by not collecting information, Answering the communities questions about HIV Prevention, Q and A section, Private chat box with providers, Joining with and connecting users to local HIV services, Establishing credibility by promoting in trusted spaces, Partnering with healthcare influencers |
| No Name at the Designathon | Young people aged 18-24 years old at St. Louis University can be expanded to a larger community at future research times | Focusing on the social-ecological model, Peer mentorship as peers are highly influential and increase trust, how we maintain behavior post-intervention, Peer mentors being trained by experts, Increasing HIV tests, Condom usage, and HIV prevention knowledge, Linking patients to care, Partnering with organizations and clubs at Saint Louis University, Patients receiving informative counseling |
| Project Angels | Young people aged 13-18 in St. Louis | HIV resources in St. Louis are not targeted towards youth; teaching youth about HIV prevention through art, Dancing, singing, painting, and all other types of art, Angels in the program will graduate and gain their halo, Exposing students to experiences of people with HIV, Expanding access to informative art to more age groups, Using peers to recruit more students, Preventing HIV by exposing students to HIV Knowledge and stores |
| HIVE - The Art of Coming Together | High School Students 14-19 | Hive the art of coming together, Targeting High school students at the individual community and institutional level, Targeting schools with demographics at risk of HIV, Designing for User-friendliness and compatibility, Adapting with continuous impact, Allowing students to access HIV Prevention knowledge, Partnering with schools to promote HIV knowledge, Empowerment of Stakeholders, Promoting fairness by providing underserved communities with vital knowledge, Creating a one-stop shop for resources, Making information accessible and clear. |
| Change is not Star Away | College-aged black women at Georgia State University | Change is Star Away; despite making up 13% of the female population, Black women make up 58% of new HIV diagnoses. Shifting the focus and creating new resources for trans women, Destigmatize HIV prevention for Black college-aged women, Using the power of social media, Providing resources and connections to community members, Partnering with local organizations, Culturally engaging information, Focusing on relationships, Ensuring participants feel “heard,” Engaging with participants where they already are, Creating an environment where people feel comfortable and welcome, Utilizing self care |
| Nulage: New Understanding and Learning in AIDS and Gender Education | Minority youth: High Schoolers aged 13-17 and trained mentors aged 18+ in Bryan/College Station, Texas | Nulage – Stigmatization in the educational setting, New understanding and learning in AIDS, Branding the HIV Topic, Normality comes from those around us, Peer-to-Peer trust and relatability, Peer mentos creating a trusting relationship, Collaborating with schools, Staying local, Creating a safe space for students, Promoting participation through Points, Partnering with local businesses |
| No Name at the Designathon | Minority youth aged 13-18 in Bryan/College Station, Texas | Focusing on: Advocacy, training, outreach, prevention dissemination, and implementation, 38 million people globally are living with HIV Peer-focused interventions, Training the Trainers, Spreading HIV prevention information and strategies, Disseminating information down the chain. Training students at School, Adapting to the local context in Texas, Youth should be taught to protect themselves. |

Abbreviations: ATL, Atlanta; PrEP, Pre-exposure prophylaxis; MSM, men who have sex with men

**Appendix 4. Judges Scores on Teams Solutions at the Innovation Bootcamp Final Pitch**

| **Solution Name** | **Judge #1** | **Judge #2** | **Judge #3** | **Judge #4** | **Judge #5** | **Judge #6** |
| --- | --- | --- | --- | --- | --- | --- |
| Tech and media leverage for PrEP uptake among MSM of color | 47 | 43 | 46 | 40 | 40 | 46 |
| Living Reality | 42 | 36 | 40 | 31 | 31 | 42.5 |
| Project SPARK | 46 | 38 | 43 | 34 | 34 | 42.5 |
| ATL in ATL | 44 | 41 | 45 | 43 | 43 | 47 |
| HIVE | 49 | 41 | 43 | 41 | 41 | 49 |
| NULAGE | 46 | 40 | 43 | 40 | 40 | 45.5 |
| Project Angels | 47 | 34 | 44 | 41 | 41 | 44 |

**Appendix 5. Additional Reports on Participants Experience with the STAR Program**

**Mentorship Access**

*(Q: Q22 Do you currently have access to a mentor that meets your professional needs?)*

**Mentorship Experience**


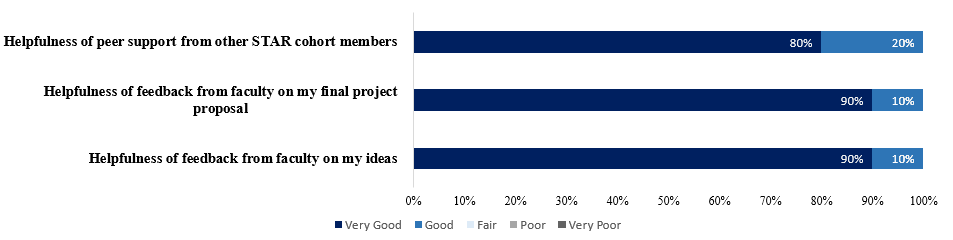


**Overall Satisfaction with the STAR Program**

*Q: How satisfied were you with the STAR program overall?*

**Recommend the STAR Program**

*Q: How likely are you to recommend STAR to colleagues?*
